# Case of Complete Intestinal Obstruction Caused by the Appendix Vermiformis in the Femoral Canal; Operation and Recovery

**Published:** 1882-07

**Authors:** H. B. Knowles

**Affiliations:** Calais, Maine


### CLINICAL REPORTS.

### CASE OF COMPLETE INTESTINAL OBSTRUCTION CAUSED BY THE APPENDIX VERMIFORM IS IN THE FEMORAL CANAL; OPERATION AND RECOVERY.

BY H. B. KNOWLES, M.D., CALAIS, MAINE

On February 11th, 1881, I was called to see a man about 65 years of age, painter by trade.—Scotch by birth ; he had always been very healthy during life (as he told me) with the exception that of late years he had been subject to occasional attacks of colic, attended with quite severe pain, nausea and vomiting. Pain in all of the attacks ; nausea and vomiting always ceased after a free evacuation of the bowels. The pain was always confined to the right iliac region. In the attack for which I was called to see him he did not get any relief from the employment of the means he had formerly in such attacks. Previous to my arrival the attending physician had given him a cathartic followed by enemas ; which, as the doctor informed me, had acted freely, and I found the patient comparatively comfortable. But the case, I think, (as the sequel will show) was mostly attributable to the effect of the hypodermic injections of morph. sulph. which the doctor had given him before my arrival. Probably the action of the bowels, of which they told me, emptied the transverse and descending colon only. This was about 12 M., and he remained quite easy the remainder of the night. But the next morning about six the pain returned, more severe than ever, accompanied by vomiting of fecal matter. Pulse 130. I was satisfied that there was intestinal obstruction somewhere in the vicinity of the ileo cecal valve, and perhaps at the valve, but could not determine that. I should have mentioned before that on my arrival the attending physician called my attention to a small tumor situated in the right femoral region ju t below Poupart's ligament. It was about the size of an English walnut. The doctor had diagnosed the case as one of strangulated femoral hernia, and had endeavored to reduce it, but failed. Upon examination of the tumor I felt positive that no intestine was involved ; neither could I satisfy myself that it was omentum. The next questions that would naturally present themselves would be, Was it an enlarged gland ? or was it a tumor ? At last I came to the conclusion that this tumor must have something to do with the intestinal obstruction, and proposed to operate, as giving the *only* chance for relief. After telling the patient and friends what I proposed to do, I proceeded to operate the same as for strangulated femoral hernia. Making an incision nearly three inches in length parallel to Poupart's ligament, I united it with a short vertical one. I then dissected up the flap formed by the incision, and cautiously divided the remaining tissues on a director. The tumor was soon exposed. It presented a dark mahogany color, smooth and shining. Upon attempting to pass my finger into the canal and around the tumor, found it quite immovable from adhesions (old). I then proceeded to break them up with the finger and handle of the scalpel, which I did, and was soon able to pass my finger around the tumor and into the abdominal cavity. There was no doubt now about its attachment to some portion of the intestines in the vicinity of the ileo-cecal valve. The question now naturally presented to my mind was, what shall I do with it ? return it to the abdominal cavity ? (which I could have easily done), or ligate and excise it ? Upon a careful examination it did not present a healthy appearance, therefore I placed a strong carbolized silk ligature around it as near as possible to its attachment, and removed it, and it proved to be the greater portion of the appendix. It proved to be glandular in structure, and hollow, as could easily be seen after making a longitudinal incision through it. I am digressing somewhat here, but will give results of the operation. Closed the wound by interrupted sutures, dressed it in the usual manner with antiseptic dressing. In three or four hours after the operation the patient had quite a *free* action of bowels ; pain, nausea and vomiting ceased, and did not return, and he made a rapid and permanent recovery. In about two weeks the wound was entirely healed, having suppurated scarcely any. The ligature came away at the twentieth day, giving no trouble. Now there can be no doubt in this case that the appendix was *the* cause of the intestinal obstruction. But in what particular way it caused the obstruction I am not prepared to say, having never seen or read of a similar case before. The fact that up to the very moment of the operation the pain, nausea and vomiting continued, but after the operation he got immediate and permanent relief, is positive proof to my mind that the appendix was the cause of the obstruction. I should mention that this man in three weeks resumed his occupation (painter). He has had none of those occasional attacks of colic which he formerly had. And this is further evidence that the operation removed the difficulty entirely.

